# Spinal Subdural Hematoma Migration From a Cranial Subdural Hematoma: Two Case Reports and Literature Review

**DOI:** 10.7759/cureus.26028

**Published:** 2022-06-17

**Authors:** Mamiko Sakai, Kensuke Hotta, Ko Ikuta, Yasuharu Nakashima

**Affiliations:** 1 Department of Orthopaedic Surgery, Kyushu Central Hospital, Fukuoka, JPN; 2 Department of Orthopaedic Surgery, Amagi Central Hspital, Asakura, JPN; 3 Department of Orthopaedic Surgery, Karatsu Red Cross Hospital, Karatsu, JPN; 4 Department of Orthopaedic Surgery, Kyushu University Hospital, Fukuoka, JPN

**Keywords:** conservative treatment, migration, cranial subdural hematoma, spinal subdural hematoma, hematoma

## Abstract

Spinal subdural hematoma (SSDH) associated with cranial subdural hematoma (CSDH) is considered extremely rare and the etiology remains unclear. Herein, we report two cases of spontaneous SSDH concomitant with CSDH, with no history of trauma. First, a healthy 35-year-old woman suffered from left leg pain following a headache caused by acute CSDH. Magnetic resonance imaging (MRI) of the lumbar spine showed SSDH extending from the L5 to S2 vertebral levels. The leg symptoms were gradually relieved with conservative treatments within two weeks after onset. The SSDH was completely resolved six months after onset on MRI evaluations. Next, a 69-year-old woman developed a headache and right hemiparesis. Brain computed tomography (CT) demonstrated chronic left-sided CSDH and she underwent a single burr-hole craniotomy. Three weeks after surgery, she experienced difficulty walking because of severe leg pain caused by SSDH extending from the L3 to S1. The clinical symptoms were completely resolved with conservative treatment within one month after onset. At 3 months follow-up, SSDH disappeared on MRI evaluation. Herein, we presented two cases of SSDH associated with CSDH. In both cases, the leg symptoms of SSDH developed following the onset of CSDH. Given that both patients remained active during the interval between CSDH onset and the appearance of SSDH symptoms, the SSDH was likely caused by migration of the CSDH contents to the lumbar spine because of gravity.

## Introduction

Spinal subdural hematoma (SSDH) associated with cranial subdural hematoma (CSDH) is considered extremely rare. Furthermore, the etiology and pathogenesis remain unclear. Previous reports have suggested etiologies involving hematoma migration from the cranial to the spinal sites or a coincidence of both CSDH and SSDH [1,2]. To our knowledge, 43 cases of simultaneous CSDH and SSDH occurrence have been reported [1-7]. Of these, only 15 cases were considered to have had both CSDH and SSDH without specific triggers [3-5]. Herein, we report two cases of spontaneous SSDH associated with CSDH.

## Case presentation

Case 1

**Figure 1:**
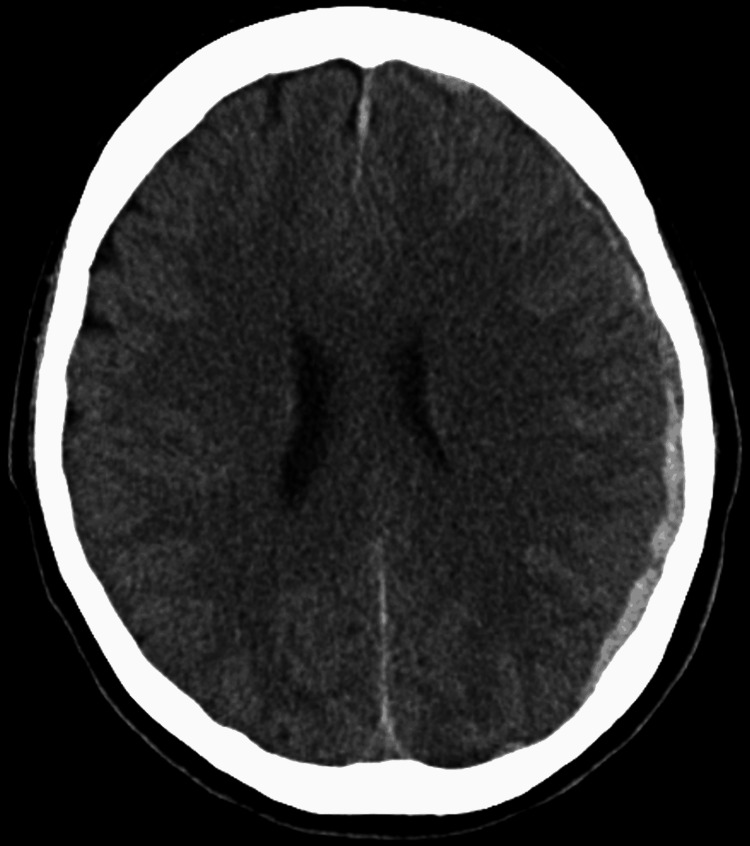
Brain CT at initial examination

A healthy 35-year-old woman, with no history of trauma, experienced headaches and saw a local doctor. Initial brain computed tomography (CT) showed no problems. However, seven days later the headaches worsened, and left buttock to posterior thigh pain appeared. Brain CT revealed left acute CSDH and she was referred to our hospital (Figure 1). Physical examination demonstrated a good general condition and neurological findings revealed no abnormalities. Laboratory analysis of coagulation and the fibrinolytic system were within reference ranges.

**Figure 2:**
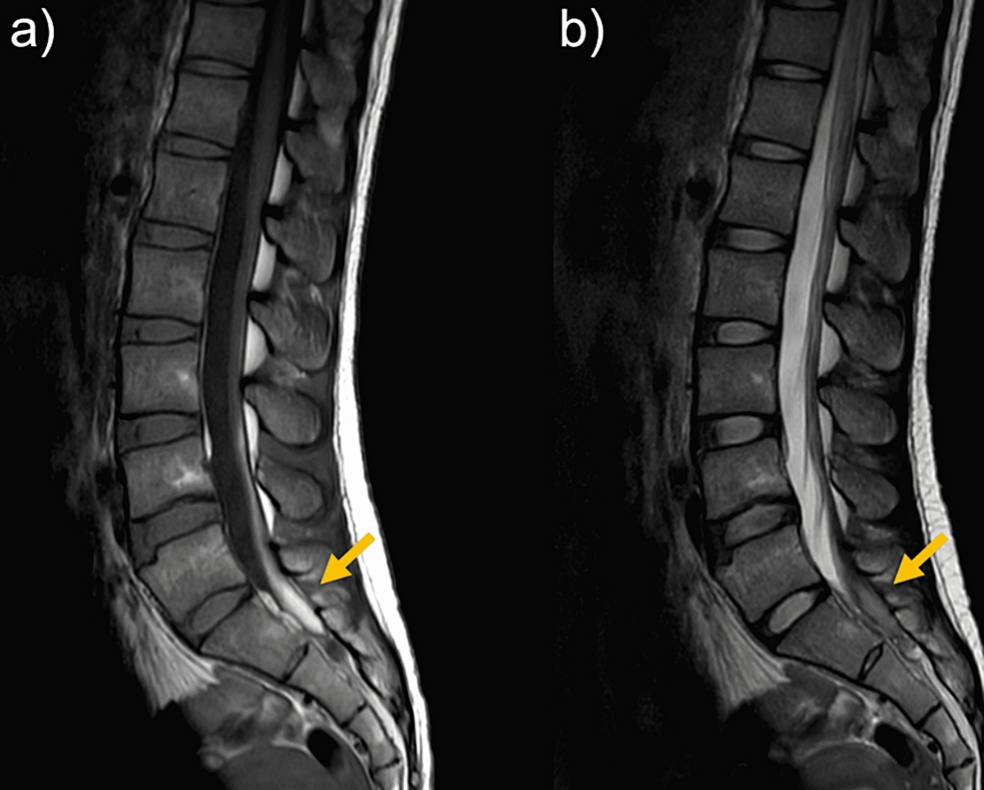
Lumber MRI at seven days after left leg pain appeared (a) Diffuse hematoma-like mass with a high signal on T1-weighted imaging. (b) An isointense signal on T2-weighted imaging

Since left leg pain persisted after admission, magnetic resonance imaging (MRI) of the lumbar spine was performed at seven days after admission. MRI showed a diffuse hematoma-like mass with a high signal on T1-weighted imaging (T1WI) (Figure 2a) and an isointense signal on T2-weighted imaging (T2WI) (Figure 2b) extending from the L5 to S1 vertebral levels in the dura mater. Additionally, a linear structure with the same intensity was observed in the frontal part of dural sac at the L1-L4. The patient was diagnosed with SSDH. The SSDH encroached the left S1 nerve root on MRI and the finding could correspond with the region of lower extremity pain. She had no symptoms of paralysis or muscle weakness and was treated conservatively.

**Figure 3:**
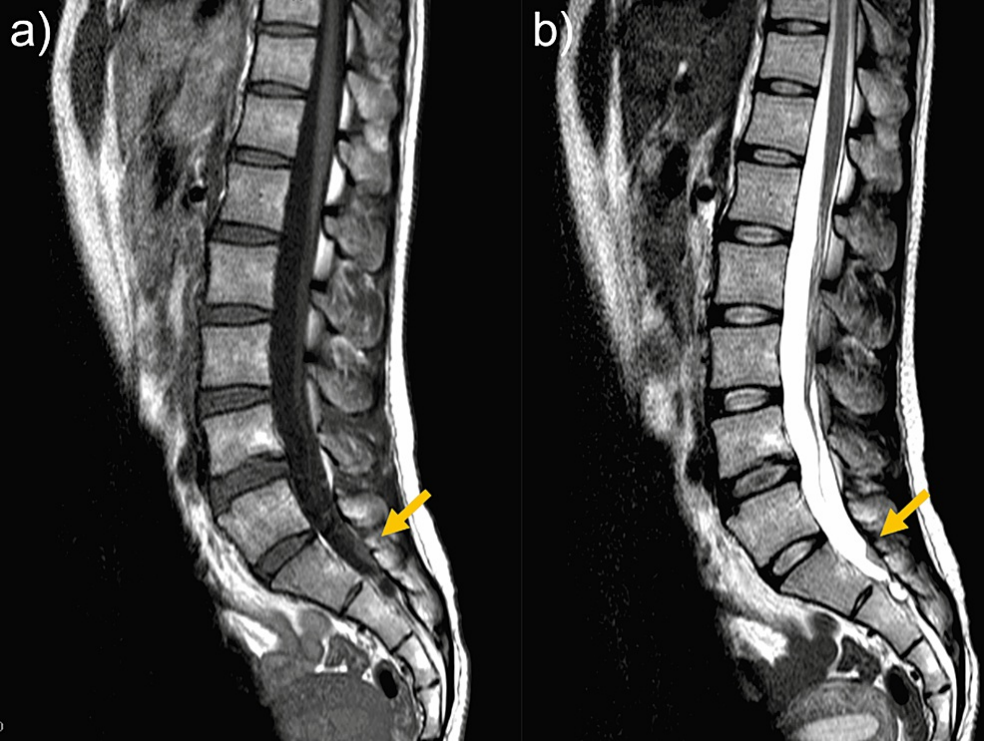
MRI at 1.5 months after onset (a) T1-weighted imaging (T1WI). (b) T2-weighted imaging (T2WI)

**Figure 4:**
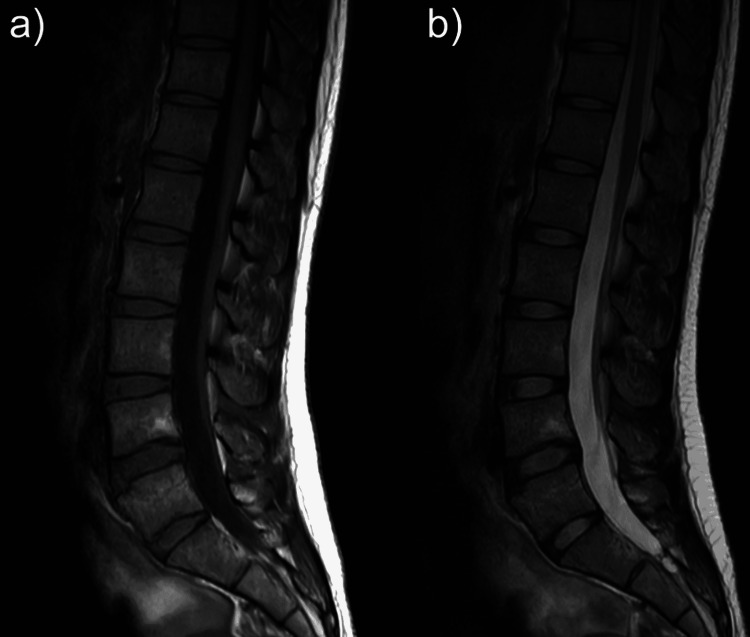
MRI at six months after onset (a) T1-weighted imaging (T1WI). (b) T2-weighted imaging (T2WI)

Three weeks after onset, headache was relieved and CSDH had disappeared on brain CT. At 1 month after onset, her leg pain was relieved. MRI obtained at 1.5 months after onset showed partial resolution of the mass, which was only located in the S1 vertebral level (Figures 3a, 3b). At the same time, the linear structures in the frontal of dural sac at the L1-L4 level also had resolved. At six months after onset, MRI showed complete resolution of the mass (Figures 4a, 4b).

Case 2

**Figure 5:**
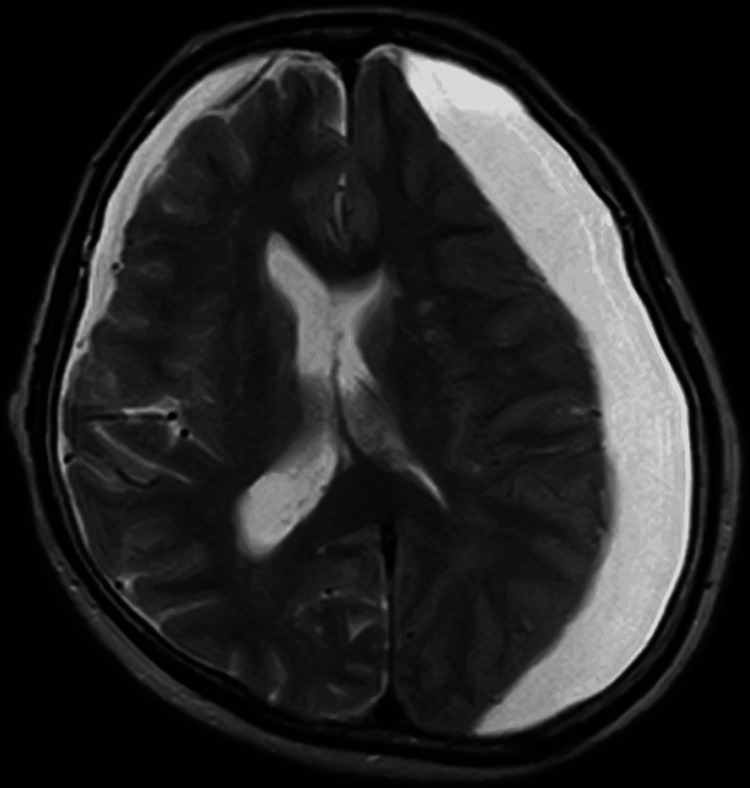
Brain MRI on admission

A 69-year-old woman with a history of hypertension and type 2 diabetes mellitus experienced headaches without any inciting cause. She visited a local doctor one month later. Brain CT demonstrated chronic CSDH bilaterally and she was prescribed Wu-ling-san. One month later, she developed a headache and slight right hemiparesis and was referred to neurosurgery. She had no history of trauma. On admission, brain MRI showed a left-sided chronic CSDH with a midline shift (Figure 5). Neurological examination showed no abnormality except for the right hemiparesis. Laboratory examinations did not indicate coagulopathy-related diseases. Evacuation of the chronic CSDH was performed by single burr-hole craniotomy on the day of admission.

**Figure 6:**
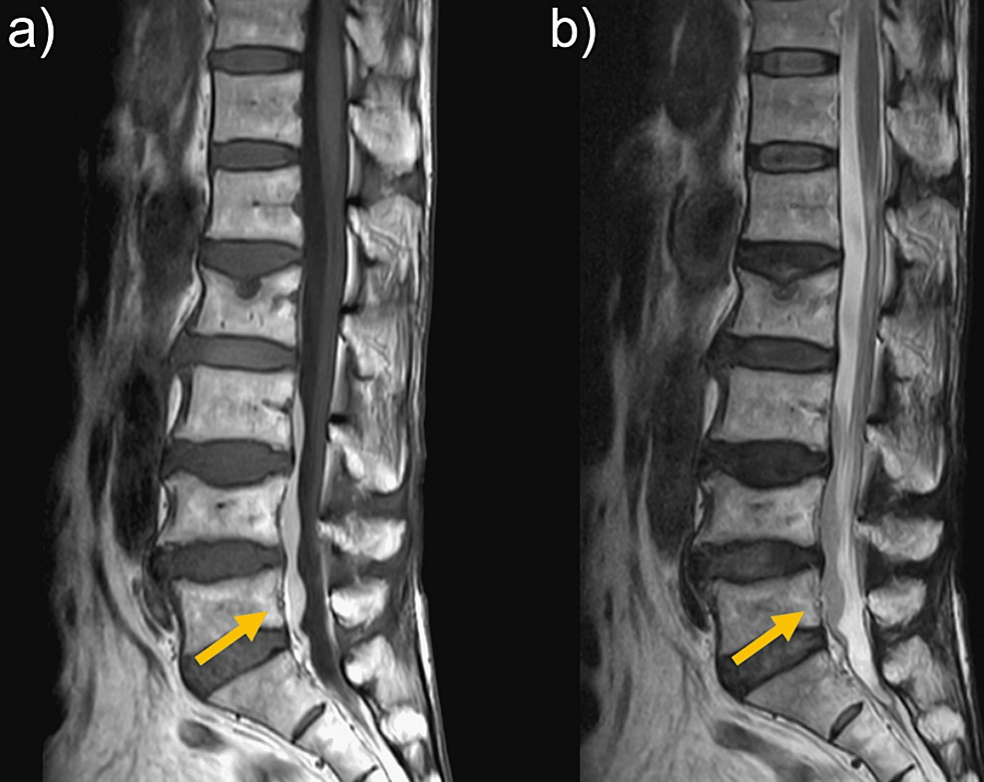
Lumber MRI at seven days after onset (a) A hematoma-like mass with a high signal on T1WI. (b) An isointense signal on T2WI.

**Figure 7:**
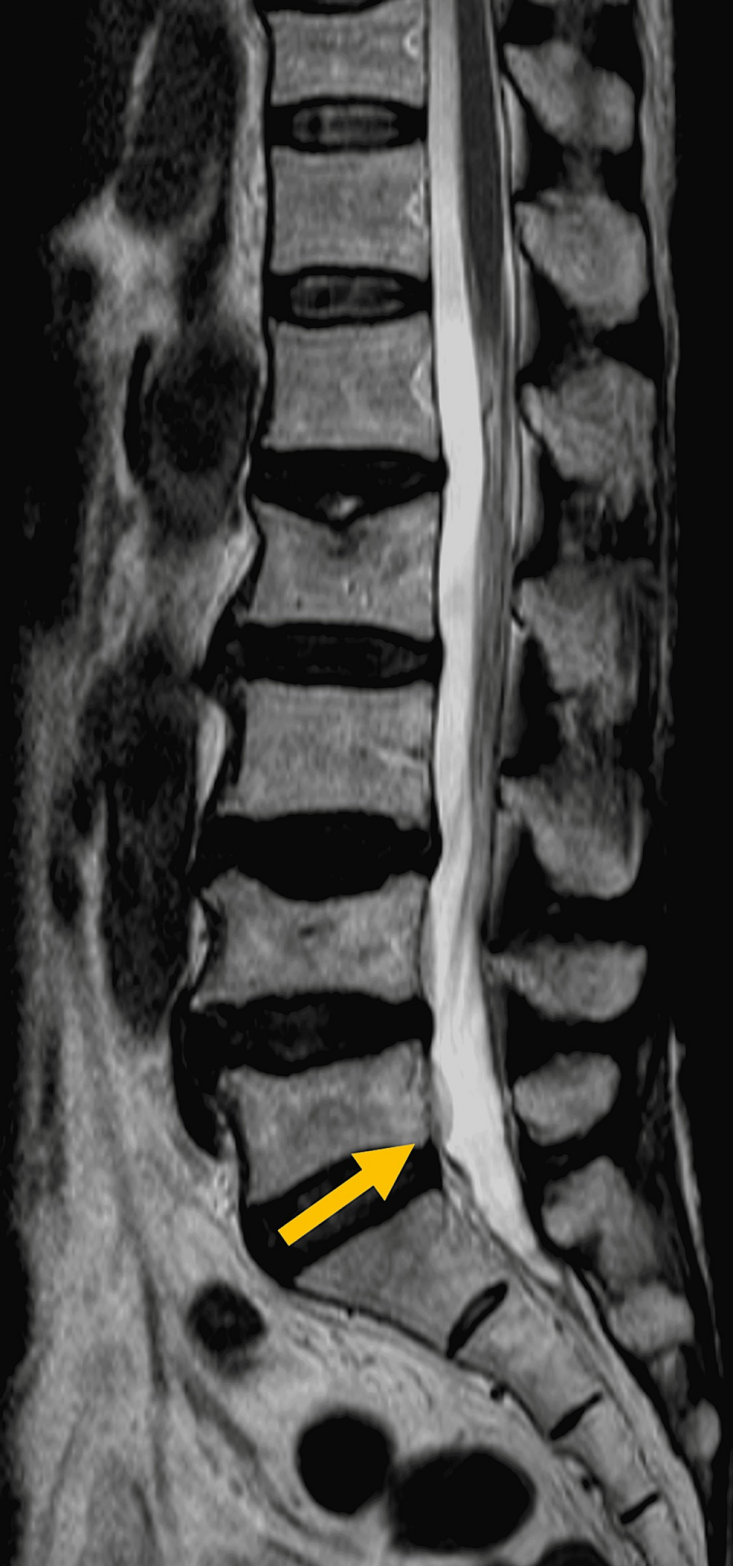
Lumber MRI at one month after onset

Although her right hemiparesis improved postoperatively, she developed left thigh pain. On lumbar MRI at seven days after onset, a hematoma-like mass with a high signal on T1WI (Figure 6a) and an isointense signal on T2WI (Figure 6b) was found extending from the L3 to S1 vertebral levels in the dura mater. The findings on MRI were not contradict the cause of the left L4 radiculopathy. The leg symptoms were relieved with conservative treatments within one month and MRI showed partial resolution of the mass, which extended from the L4 to S1 vertebral levels (Figure 7).

**Figure 8:**
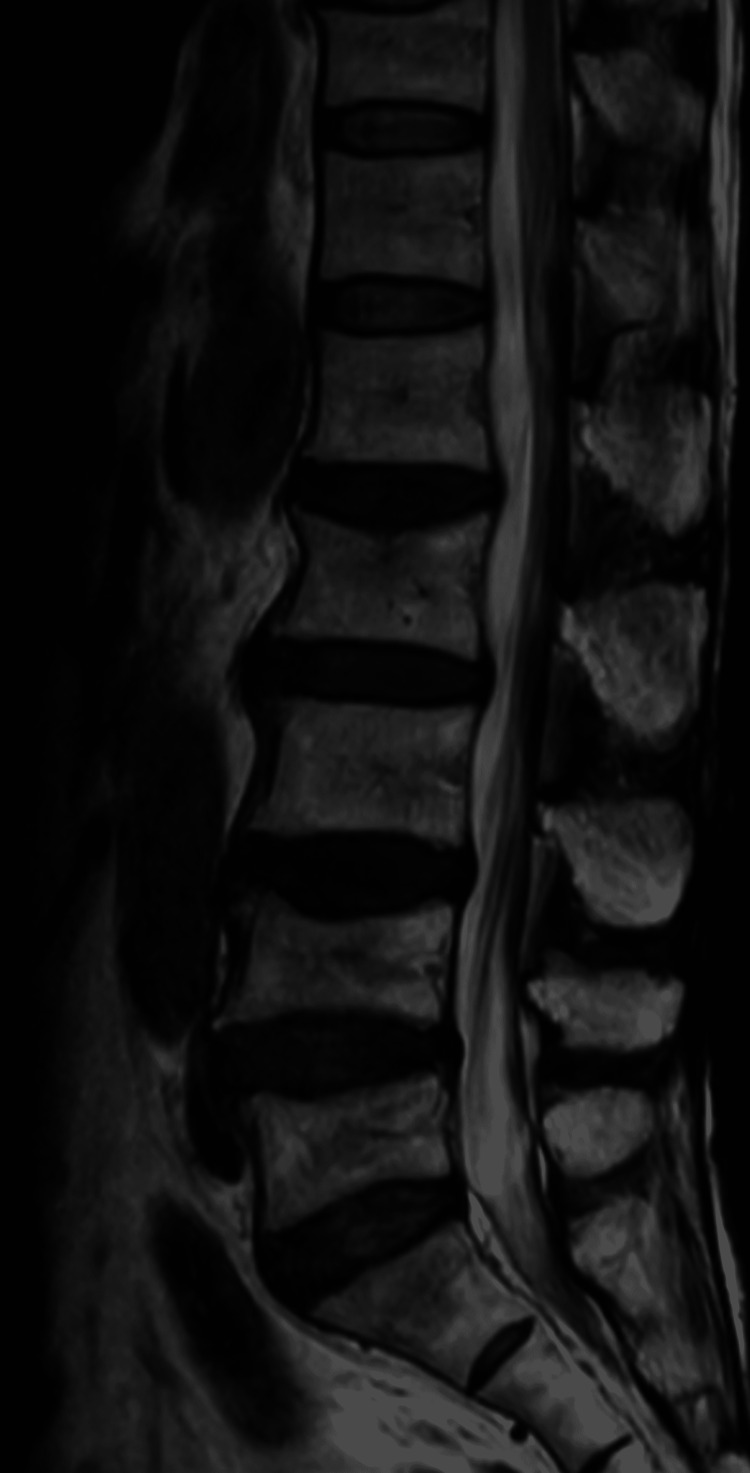
Lumber MRI at three months after onset

At three months after onset, MRI showed complete resolution of the mass (Figure 8).

## Discussion

Although SSDH itself is a rare condition, SSDH associated with CSDH is extremely rare and its etiology remains unclear. To the best of our knowledge, 43 cases of simultaneous CSDH and SSDH cases have been reported. Of these cases, 22 were post-trauma, three were post-craniotomy, three were after ventriculoperitoneal shunt placement, and the remaining 15 were spontaneous cases [1-7].

In general, the pathogenesis of SSDH is related to anti-coagulation, blood dyscrasia, vascular malformation, low cerebral spinal fluid pressure, traumatic injuries, lumbar puncture, and spinal anesthesia [3]. In our two cases, a hematoma-like mass was found in the lumbar subdural region. On the basis of this finding alone, we could not absolutely confirm that the mass was a hematoma. Adipose tissue and hematoma are both considered contents of the mass. For adipose tissue, MRI shows a high signal on both T1WI and T2WI, and it is unlikely that it would be absorbed naturally. In our cases, MRI showed a high signal on T1WI and an isointense signal on T2WI, and the mass spontaneously disappeared over time. On the basis of these findings, we considered that the mass was a hematoma and that the SSDH may be associated with CSDH.

Two mechanisms of SSDH development associated with CSDH have been reported. The first involves the migration of the hematoma from the cranial to the spinal compartments [1,2]. The abnormal intracranial pressure associated with CSDH was proposed as a predisposing factor. For example, Matsumoto et al. reported that raised intracranial pressure may displace the hematoma to the skull base or spinal canal [4]. On the other hand, low intracranial pressure resulting from a ventriculoperitoneal shunt may cause dissection between the dura and the arachnoid layer. Both high and low intracranial pressure may be contributing factors to hematoma migration. Thus, hematoma migration is under the influence of gravity. It was also suggested that progressive CSDH growth may cause a tear of the inner membrane, resulting in hematoma leakage into the subdural space and hematoma migration into the spinal subdural space under the influence of gravity [4]. The second mechanism is a coincidental occurrence of both CSDH and SSDH. In such cases, the patients may have hit both their head and back, resulting in minor trauma and accidental CSDH and SSDH [4].

Neither of our cases had a history of trauma. Nevertheless, leg pain occurred in both cases after their headaches worsened, indicating that there was a time lag between the onset of headache and leg pain. Moreover, both cases were active without any activities or daily living restrictions after headache onset. It is possible that the hematoma grew when the headache worsened and that the SSDH was caused by the migration of the CSDH contents to the lumbar spine because of gravity. Additionally, in both cases, the hematoma was located in the lower lumbar to the sacral vertebrae, which was predominantly on the tail side. Furthermore, in case 1, the SSDH at the L5-S1 level and a subdural hematoma-like lesion at the L1-L4 level had resolved spontaneously in the same period. These findings also support a mechanism involving hematoma migration from the cranial to the spinal compartments because of gravity.

Of interest, Kokubo et al. reported that 1.2% of CSDH patients showed concomitant SSDH and had asymptomatic SSDH [5]. Thus, silent concomitant CSDH and SSDH may be more prevalent than currently considered. Future case reports will help clarify this disorder.

## Conclusions

Herein, we presented two cases of SSDH associated with CSDH. SSDH associated with CSDH is extremely rare and should be suspected in CSDH patients who develop lumbago and neurologic leg symptoms. There is also potential for the hematoma to migrate from the cranial to the spinal space compartments, particularly if the CSDH patients are active. Doctors should be aware of these conditions and check neurological signs cautiously.
